# Supplementary material for: Luteolin-3′-O-Phosphate Inhibits Lipopolysaccharide-Induced Inflammatory Responses by Regulating NF-κB/MAPK Cascade Signaling in RAW 264.7 Cells
Source: Molecules. 2021 Dec 6;26(23):7393. doi: 10.3390/molecules26237393 (PMC8659157; doi:10.3390/molecules26237393)

Table S1: ^{1}H (DMSO-D6, 500 MHz NMR) and ^{13}C (DMSO-D6, 125 MHz NMR) NMR chemical shifts of Luteolin 3'-*O*-phosphate (LTP)

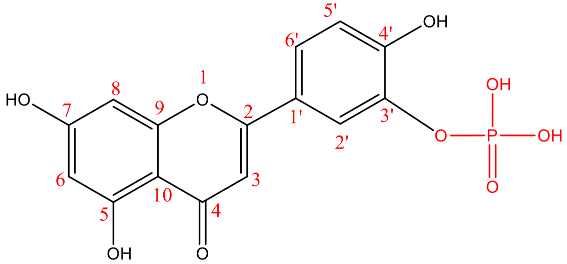

Luteolin-3'-*O*-Phosphate
Exact Masas : 366.01

No.	Luteolin			Luteolin-3'-*O*-phosphate		
	^{1}H		^{13}C	^{1}H		^{13}C
2			164.1			163.81
3	6.67	s	103.4	6.73	s	103.68
4			181.7			182.29
5			161.4			162.1
6	6.2	d(2.0)	98.8	6.2	d	99.49
7			163.8			164.89
8	6.45	d(2.0)	94.8	6.47	d	94.5
9			157.2			157.8
10			103.6			104.3
1'			121.9			121.7
2'	7.4	d(2.0)	113.9	7.71	s	120.28
3'			145.8			141.47
4'			149.7			153.93
5'	6.88	d(8.4)	114.9	6.97	d	118.67
6'	7.43	dd(2.0,8.4)	118.6	7.69	d	123.83
5-OH	12.98	s		12.94		
7-OH	10.72	s		10.9		
3'-OH	9.68	brs 2H				
4'-OH	9.68	brs 2H				

Figure S1: ^{1}H NMR spectra of Luteolin 3'-*O*-phosphate (LTP)

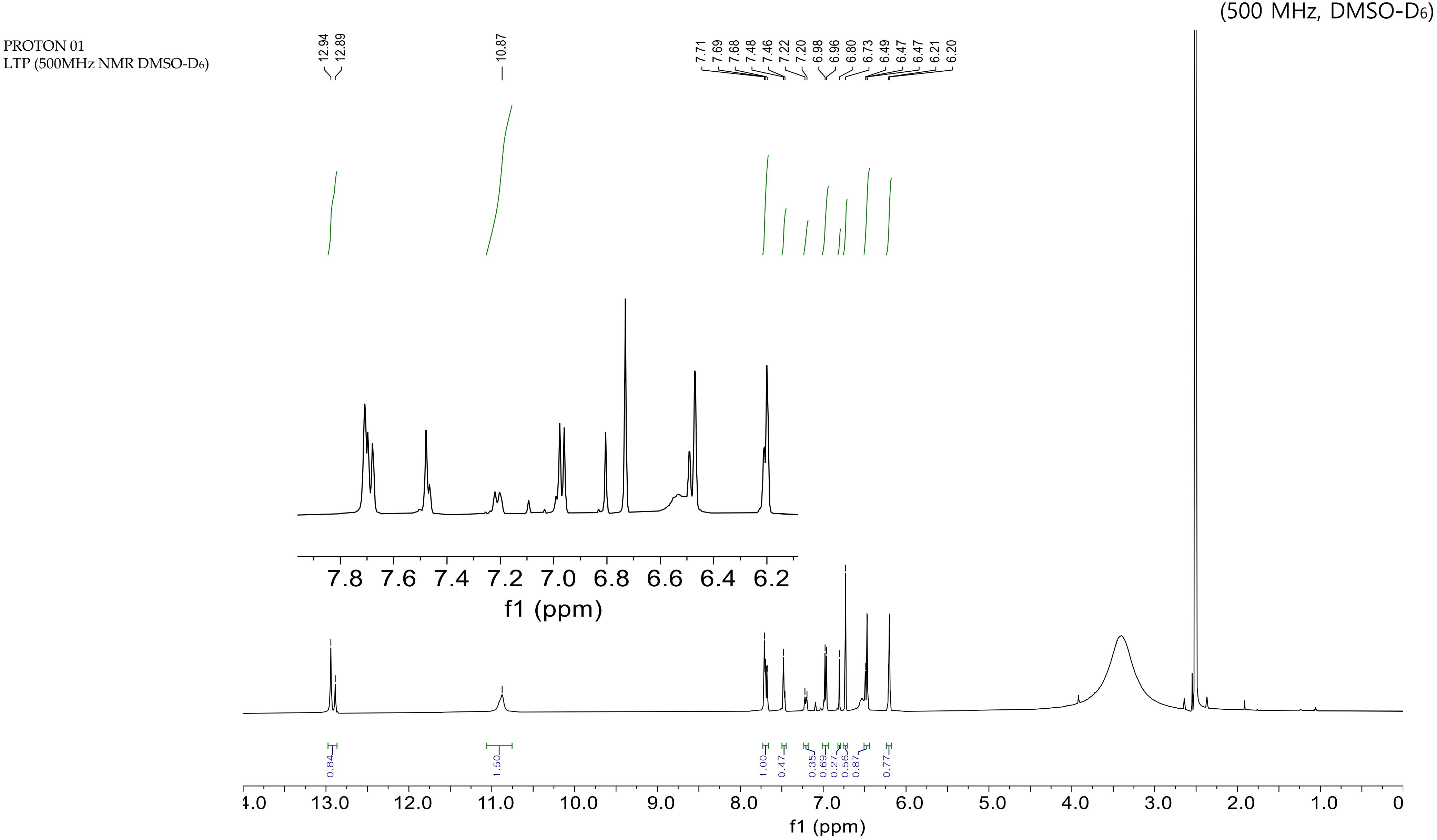

Figure S2: HSQC correlation of Luteolin 3'-*O*-phosphate (LTP)

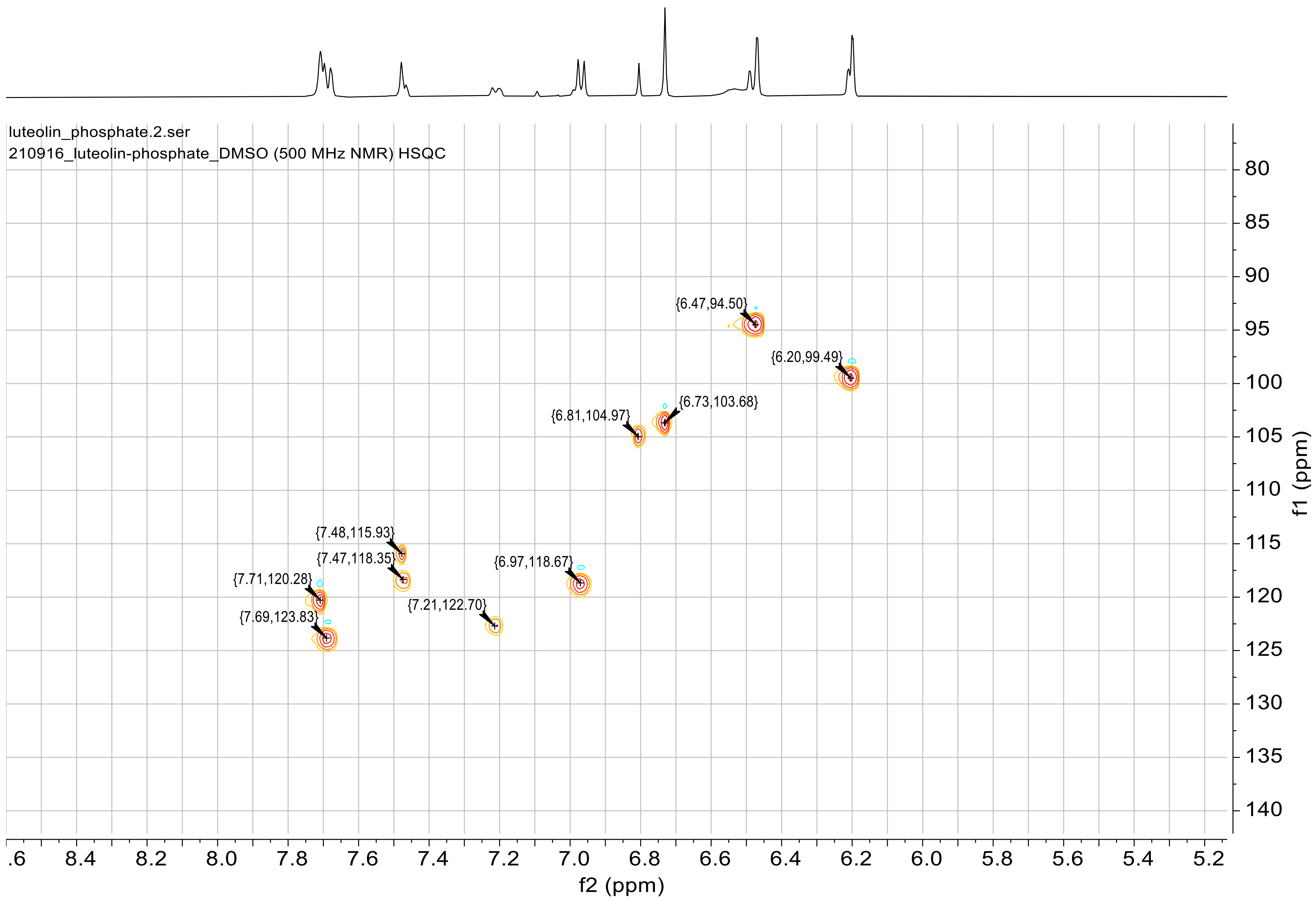

Figure S3: HMBC correlation of Luteolin 3'-*O*-phosphate (LTP)

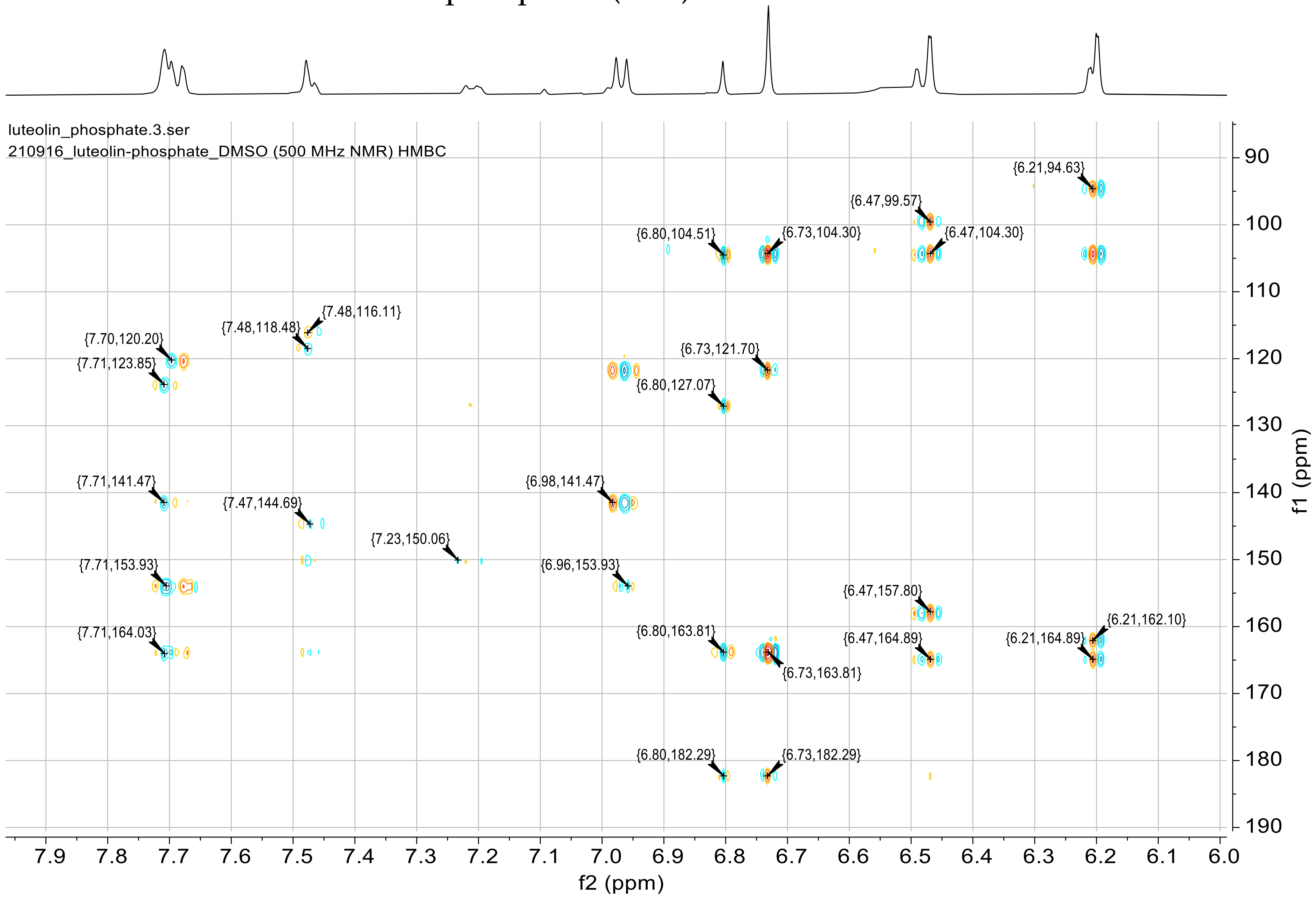

Figure S4: COSY correlation of Luteolin 3'-*O*-phosphate (LTP)

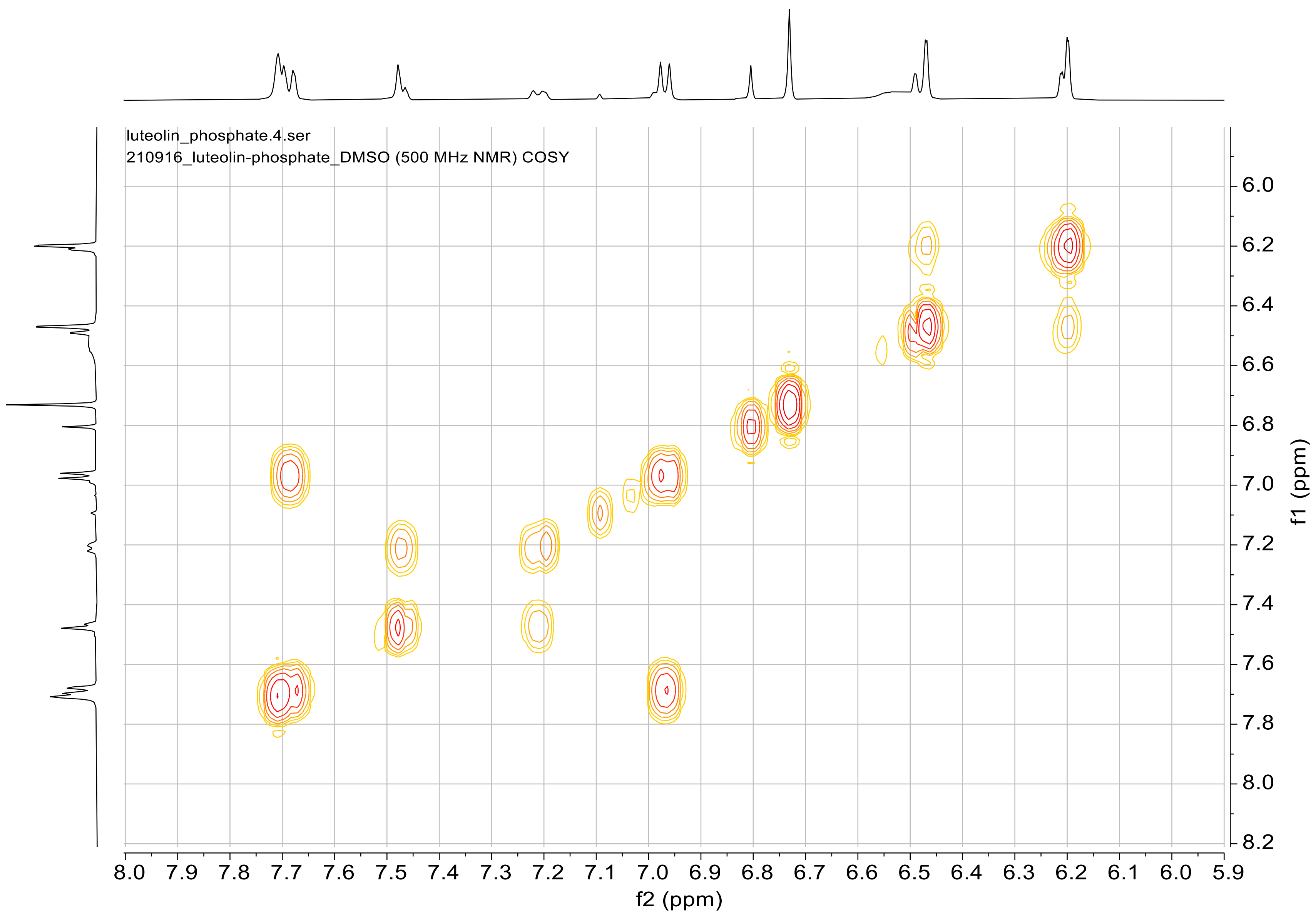

Supplement: Supplementary file 1 [file molecules-26-07393-s001.zip › molecules-1478971-supplementary.pdf]
